# Prediction of Posterior Paraglottic Space and Cricoarytenoid Unit Involvement in Endoscopically T3 Glottic Cancer with Arytenoid Fixation by Magnetic Resonance with Surface Coils

**DOI:** 10.3390/cancers11010067

**Published:** 2019-01-10

**Authors:** Marco Ravanelli, Alberto Paderno, Francesca Del Bon, Nausica Montalto, Carlotta Pessina, Simonetta Battocchio, Davide Farina, Piero Nicolai, Roberto Maroldi, Cesare Piazza

**Affiliations:** 1 Department of Radiology, University of Brescia, 25123 Brescia, Italy; marcoravanelli@hotmail.it (M.R.); c.pessina@studenti.unibs.it (C.P.); davide.farina@unibs.it (D.F.); maroldi@med.unibs.it (R.M.); 2 Department of Otorhinolaryngology—Head and Neck Surgery, University of Brescia, 25123 Brescia, Italy; delbonfrancesca@gmail.com (F.D.B.); nausica.montalto@gmail.com (N.M.); pieronicolai@virgilio.it (P.N.); 3 Department of Pathology, University of Brescia, 25123 Brescia, Italy; simonetta.battocchio@gmail.com; 4 Department of Otorhinolaryngology, Maxillofacial, and Thyroid Surgery, Fondazione IRCCS, Istituto Nazionale dei Tumori di Milano, University of Milan, 20133 Milan, Italy; ceceplaza@libero.it

**Keywords:** laryngeal cancer, magnetic resonance imaging, surface coils, posterior laryngeal compartment, cricoarytenoid unit

## Abstract

Discrimination of the etiology of arytenoid fixation in cT3 laryngeal squamous cell carcinoma (SCC) is crucial for treatment planning. The aim of this retrospective study was to differentiate among possible causes of arytenoid fixation (edema, inflammation, mass effect, or tumor invasion) by analyzing related signal patterns of magnetic resonance (MR) in the posterior laryngeal compartment (PLC) and crico-arytenoid unit (CAU). Seventeen patients affected by cT3 glottic SCC with arytenoid fixation were preoperatively studied by state-of-the-art MR with surface coils. Different signal patterns were assessed in PLC subsites. Three MR signal patterns were identified: A, normal; B, T2 hyperintensity and absence of restriction on diffusion-weighted imaging (DWI); and C, intermediate T2 signal and restriction on DWI. Signal patterns were correlated with the presence or absence of CAU and PLC neoplastic invasion. Patients were submitted to open partial horizontal or total laryngectomy and surgical specimens were analyzed. Pattern A and B did not correlate with neoplastic invasion, while Pattern C strongly did (Spearman’s coefficient = 0.779, *p* < 0.0001; sensitivity: 100%; specificity: 78%). In conclusion, MR with surface coils is able to assess PLC/CAU involvement with satisfactory accuracy. In absence of Pattern C, arytenoid fixation is likely related to mass effect and/or inflammatory reaction and is not associated with neoplastic invasion.

## 1. Introduction

**Figure 1:**
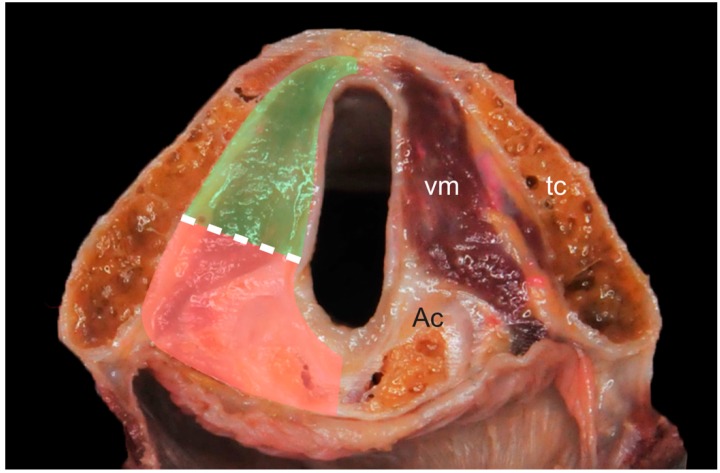
Anatomical section representing the glottic plane on a transverse section: dashed line (“magic plane” according to Succo et al. [4]) indicates the limit dividing the anterior (green) from the posterior (red) laryngeal compartments. Legend: Ac, arytenoid cartilage; tc, thyroid cartilage; vm, vocal muscle.

Endoscopic evaluation represents a paramount diagnostic and staging tool for the assessment of laryngeal tumors. Vocal fold (i.e., arytenoid) fixation in glottic squamous cell carcinoma (SCC) leads to classification in the cT3 category according to the 7th Edition of the AJCC UICC TNM staging system (unchanged in the subsequent 8th Edition) [1,2]. This clinical sign has recently been better defined by Rosen et al. [3] as “vocal fold immobility related to laryngeal malignant disease”. However, such a clinical diagnosis may either underestimate the true neoplastic extent (e.g., pT4 with clinically silent cartilage invasion or extralaryngeal spread) or overestimate it (e.g., in “simple” pT2 when the cricoarytenoid unit (CAU) is only impaired in its normal function by peritumoral edema or tumor bulk rather than by true neoplastic infiltration). On the other hand, from an etiopathological point of view, vocal fold/arytenoid immobility can be related to a number of different factors: invasion of the CAU, intrinsic laryngeal muscle involvement, infiltration of the intra- and/or extralaryngeal nerve supply, volume (mass) effect, and inflammatory changes. This leads to substantial diagnostic uncertainty, since these conditions have relevant and dissimilar clinical implications. For example, in a recently published multicentric study on T3 and T4 laryngeal SCC treated by open partial horizontal laryngectomies (OPHLs), pT3 tumors with posterior laryngeal compartment (PLC) invasion (i.e., the paraglottic space located posterior to a line drawn from the arytenoid vocal process perpendicularly to the thyroid cartilage, of which the CAU represents a key structure) demonstrated significantly worse prognosis than anteriorly located pT4 lesions [4]. For this reason, the theoretical plane dividing the anterior from the PLC was also called by the authors the “magic plane” (Figure 1).

Imaging has the ability to accurately delineate submucosal tumor extension in order to elucidate the cause of arytenoid hypomotility or fixation. In particular, we have defined as “arytenoid fixation” the complete immobility of both the vocal fold and arytenoid cartilage [3]. Magnetic resonance (MR) offers higher contrast resolution than computed tomography (CT) and is particularly helpful in distinguishing tumor infiltration from peritumoral inflammation, and in depicting cartilage infiltration [5]. State of the art protocols, including use of surface coils, enhance the potential of the technique by significantly increasing its spatial resolution.

The aim of this study was to explore the capability of MR in assessing the PLC, with special emphasis given to evaluation of CAU, in clinically-defined T3 SCC with arytenoid fixation at preoperative endoscopic evaluation.

## 2. Materials and Methods

### 2.1. Patient Selection

**Figure 2:**
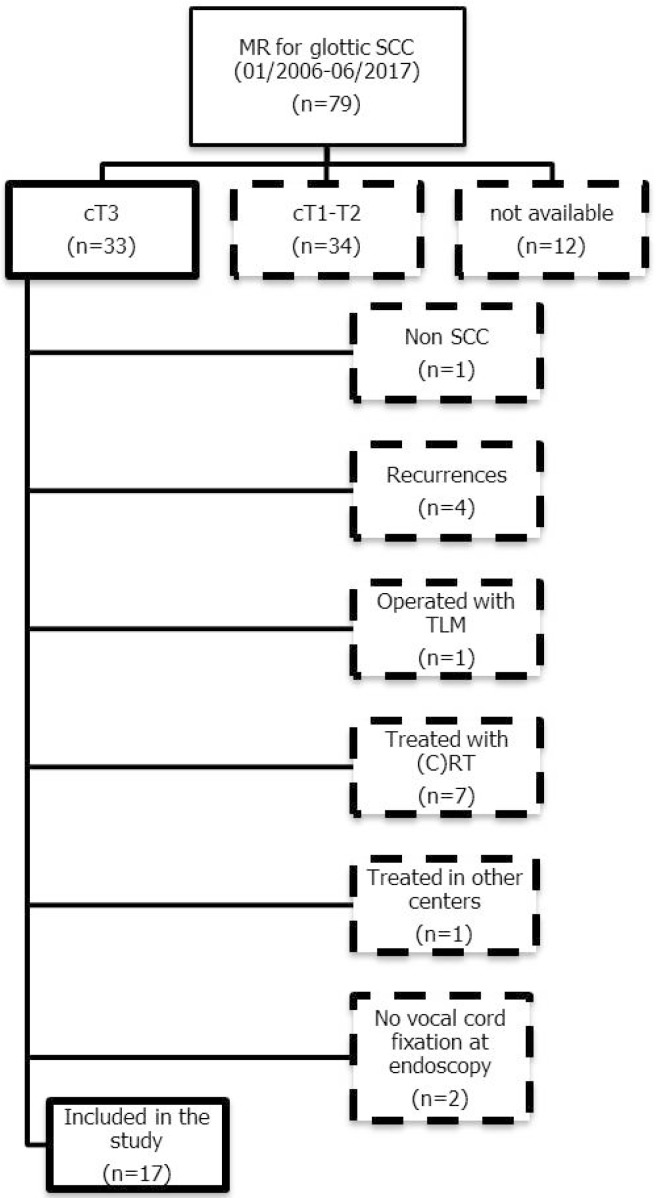
Selection schema: flow chart of patient enrollment in the present study. Legend: MR, magnetic resonance; SCC, squamous cell carcinoma; TLM, transoral laser microsurgery; (C)RT, (chemo)radiotherapy.

The present retrospective study was performed in a tertiary academic center. It included 17 patients (13 males and four females: mean age, 65 years; age range, 48–82) with histologically demonstrated glottic SCC defined as cT3 for arytenoid fixation via transnasal flexible videoendoscopy. This is a purely retrospective study with no need to receive approval from ethics committee, and no informed consent was signed. Each examination was video-recorded and separately re-evaluated by two experienced laryngologists/head and neck surgeons to confirm the clinical immobility of the vocal fold with arytenoid fixation. MR was performed in the same institution within two weeks from surgery. All patients were subsequently treated by OPHL or total laryngectomy (TL) according to clinical staging, patient comorbidities, and preference. A flow-chart of patient selection is shown in Figure 2.

### 2.2. MR Study Protocol

MR studies were performed on a 1.5T Siemens Avanto or Siemens Aera scanner (Siemens Healthcare, Erlanghen, Germany) using two small loop coils (circular coils with a diameter of 4 cm specifically designed to provide improved signal-to-noise ratios by limiting the spatial extent of the excitation and reception) directly applied to the larynx as per standard protocol in our institution. The MR protocol included high resolution TSE-T2 and TSE-T1 sequences (with slice thicknesses ranging from 2 to 3 mm and a matrix of 512 or 448 with reduced field of view) which were often replaced in uncooperative patients by sequences with motion-compensation (radial k-space filling) with a 320 matrix. Diffusion-weighted imaging (DWI) was acquired with monopolar gradients and two b-values (0 and 1000 mm/s^2^). After contrast injection, a 3D GE sequence with fat saturation (VIBE) was acquired with 0.6 mm voxel size; in uncompliant patients, it was replaced with a motion-compensated sequence with 0.7 mm voxel size sequence (StarVIBE).

### 2.3. Image Analysis

Images were reviewed in consensus by two experienced head and neck radiologists blinded on the videoendoscopic examination, type of surgery performed, intraoperative findings, and definitive histopathologic report. The PLC was defined as the area located behind a line drawn in the axial plane from the vocal process of the arytenoid to the ipsilateral thyroid lamina and perpendicular to the latter (Figure 1). In this compartment, the following structures were systematically assessed: the posterior portion of the vocal (medial thyro-arytenoid) muscle, the lateral crico-arytenoid and crico-thyroid muscles, the posterior portion of the paraglottic space (PGS) (i.e., the part of the PGS located into the PLC as defined above), the inferior PGS (i.e., the part of the PGS located below the vocal ligament, lateral to the conus elasticus, and medial to the cricoid cartilage), the arytenoid, and the cricoid cartilage. To assess cartilage invasion we used the criteria proposed by Becker et al. [6]. Each of these structures was categorized according to their signal pattern in the different sequences:

Pattern A: normal signal intensity in all sequences;

Pattern B: tissue changes characterized by T2 hyperintensity, no DWI restriction (high apparent diffusion coefficient (ADC)), and variable contrast-enhancement. For ossified cartilages: T2 hyperintensity, T1 hypointensity, and bright contrast enhancement;

Pattern C: tissue abnormalities characterized by T2 intermediate signal, DWI restriction (high DWI signal combined with low ADC value), and variable contrast-enhancement. For ossified cartilages: T2 intermediate signal, T1 hypointensity, and intermediate contrast enhancement.

**Figure 3:**
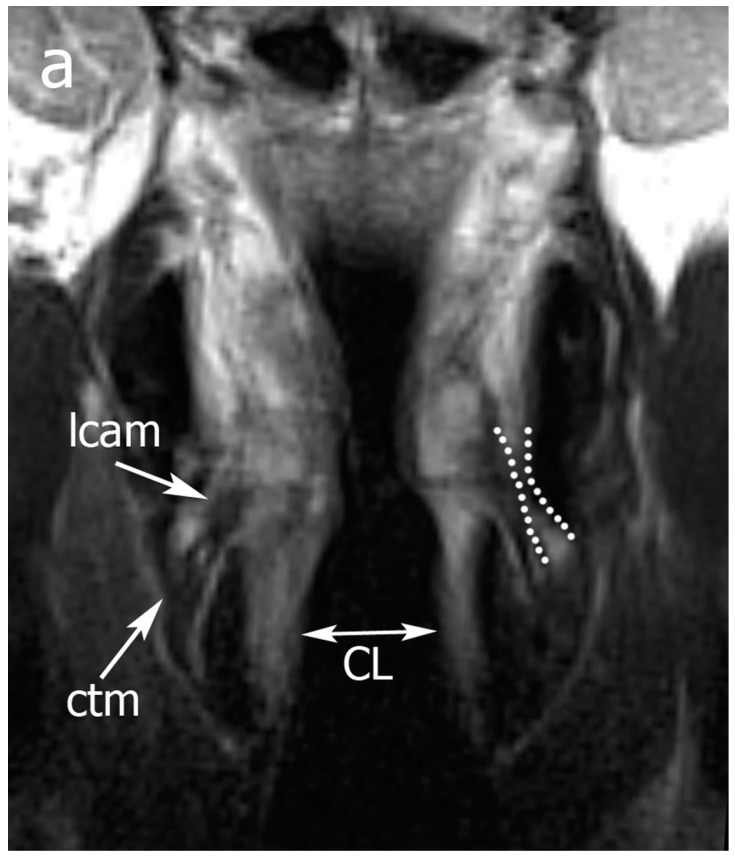

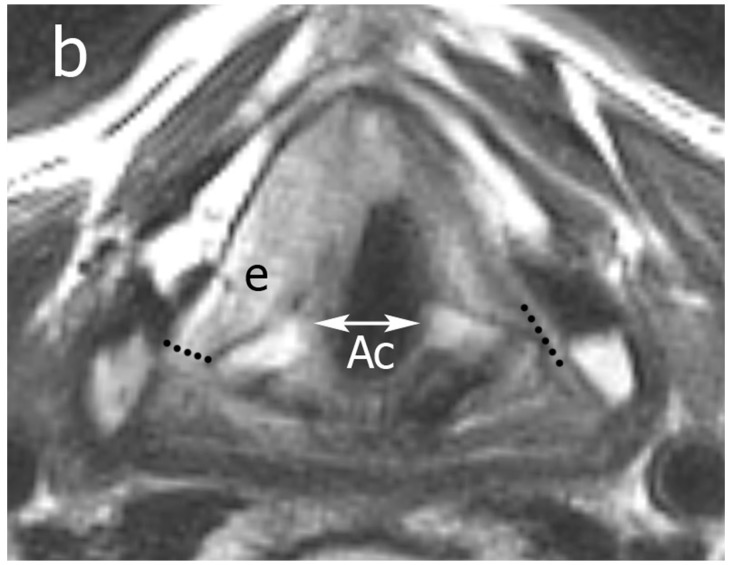
Example of Pattern B. From top to the bottom: (**a**) TSE-T2 on coronal plane; (**b**) TSE-T2 on axial plane. A right glottic tumor that does not invade the posterior laryngeal compartment (PLC) is seen. In (**a**), normal signals of lateral cricoarytenoid and cricothyroid muscles are displayed, the cricoid lamina is normal, and an inferior paraglottic space (PGS) is indicated by dotted lines. In (**b**), expansion of the right PLC (dotted black lines) is seen. A high T2 signal in the PLC is consistent with an intense edema that hampers crico-arytenoid unit (CAU) functionality. Legend: Ac, arytenoid cartilages; CL, cricoid lamina; ctm, cricothyroid muscle; e, edema; lcam, lateral cricoarytenoid muscle.

**Figure 4:**
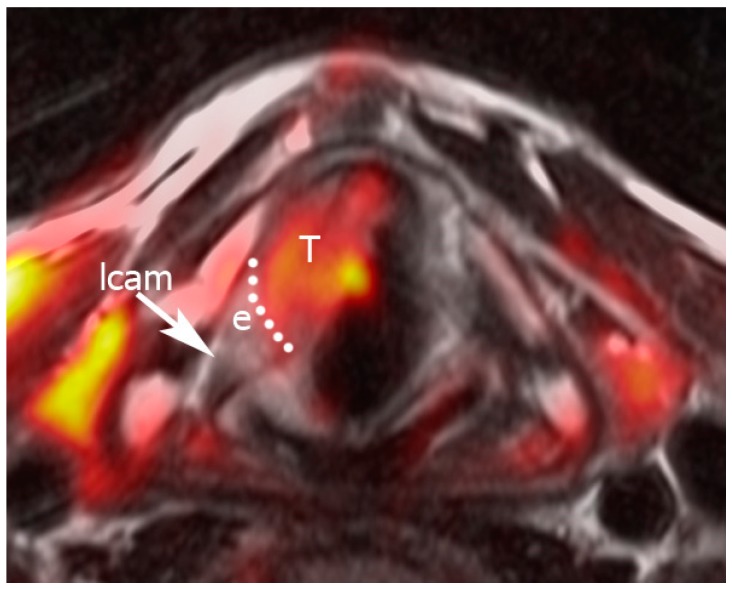
Example of Pattern B, indicating fusion between a T2-weighted sequence and a diffusion-weighted imaging (DWI) image. “Hot” areas correspond to the anterior glottic tumor. A dotted line separates the tumor from the peritumoral edema. The lateral cricoarytenoid muscle is normal. Legend: e, edema; lcam, lateral cricoarytenoid muscle; T, tumor.

**Figure 5:**
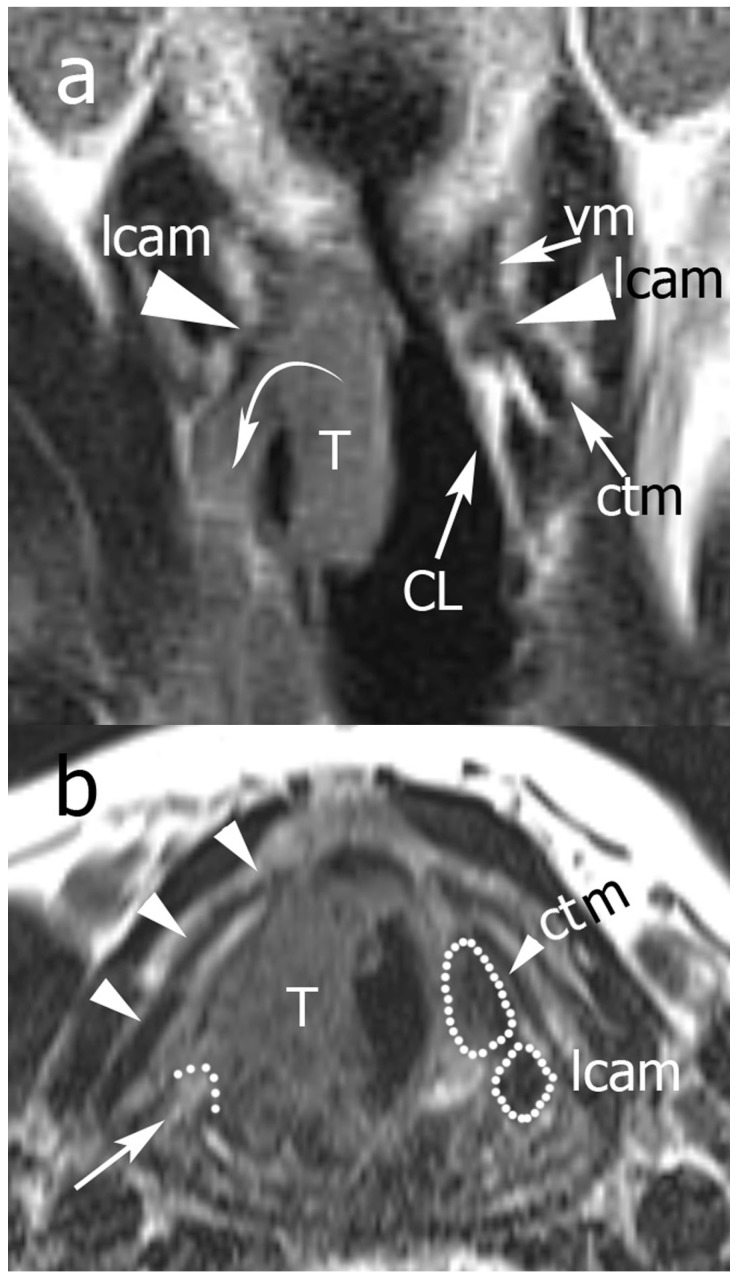
Example of Pattern C. From top to bottom: (**a**) TSE-T2 on coronal plane; (**b**) TSE-T2 on axial plane. In (**a**) the right glottic-subglottic tumor infiltrates the inferior PGS (curved arrow) extending outside the larynx, the vocal and lateral cricoarytenoid muscles are partially infiltrated, and the cricoarytenoid muscle is completely undetectable because of invasion (the same muscles are normally visible on the left side). There is infiltration of the right cricoid lamina, although it is normal on the left side. In (**b**) the tumor infiltrates the lateral cricoarytenoid and cricothyroid muscles, visible on the left side. Arrowheads indicate the right thyroid lamina, which is not infiltrated. A curved dotted line and arrow indicate the posterior front of the neoplasm. Legend: CL, cricoid lamina; ctm, cricothyroid muscle; lcam, lateral cricoarytenoid muscle; T, tumor; vm, vocal muscle.

Pattern B was considered consistent with high water content within tissues and was thus more likely to be associated with inflammatory changes (Figure 3 and Figure 4), while Pattern C was considered more likely to be consistent with tumor infiltration (Figure 5).

Scores (0 for Pattern A, 1 for Pattern B, and 2 for Pattern C) for each subsite (*n* = 7) were summed to obtain three different scores. The “Overall Score” (ranging from 0 to 14) was derived from the sum of the scores assigned to each subsite. The “Partial Score Pattern B” (ranging from 0 to 7) was derived from the sum of each subsite receiving a score of 1. The “Partial Score Pattern C” (ranging from 0 to 14) was derived from the sum of each subsite receiving a score of 2. The three scores were then correlated with specific histopathological findings as described in the statistical analysis section.

Imaging findings were compared to definitive histopathological evaluations of formalin-fixed surgical specimens after OPHL or TL: in 11 patients, the report overtly described the PLC as invaded or not. In six patients, revision of macrosections was required and performed by a dedicated pathologist since such information was missing in the original report or was not clear enough. In these cases, the criterion used to identify or rule out the PLC invasion was the presence of neoplastic cell nests posterior to the aforementioned “magic plane” (Figure 1).

### 2.4. Statistical Analysis

Spearman’s test was used to assess correlation between CAU infiltration and different MR patterns. Area Under the Receiver Operating Curve (AUROC) was used to assess if the three radiological scores had an association with histology and diagnostic performance using the best cut-off value. Statistical significance was fixed at 0.05. All statistical tests were performed in MedCalc (MedCalc Software bvba, Ostend, Belgium).

## 3. Results

### Patients and CAU/PLC Infiltration

Figure 2 gives a flow chart of patient enrollment. Table 1 summarizes the baseline characteristics of patients and the type of surgery they received, while Table 2 shows the three scores calculated for each patient. Upon definitive histopathological examination, eight (47%) patients showed involvement of the PLC. With MR, one (6%) patient of the 17 had Score 0 (related to Pattern A) in all subsites and sequences: histology confirmed no signs of PLC involvement (true negative). Six (35%) had Score 1 (Pattern B) in at least one subsite, without any Score 2 (Pattern C): histology showed no signs of PLC infiltration (true negatives). Ten (59%) patients had Score 2 (Pattern C) in at least one subsite: histology showed PLC invasion in eight cases (true positives). Two patients turned out to have false positives. In Table 2, the three MR scores per each patient are reported and compared to their histology.

Spearman’s test demonstrated no correlation between the “Partial Score Pattern B” and neoplastic involvement of PLC (−0.0124; CI 95%, −0.490–0.471; *p* = 0.9624), while the “Overall Score” showed significant positive correlation with PLC infiltration (0.690; CI 95%, 0.313–0.879; *p* = 0.002). However, the strongest correlation was found between PLC infiltration and “Partial Score Pattern C” (0.779; CI 95%, 0.477–0.916; *p* = 0.0002).

**Figure 6:**
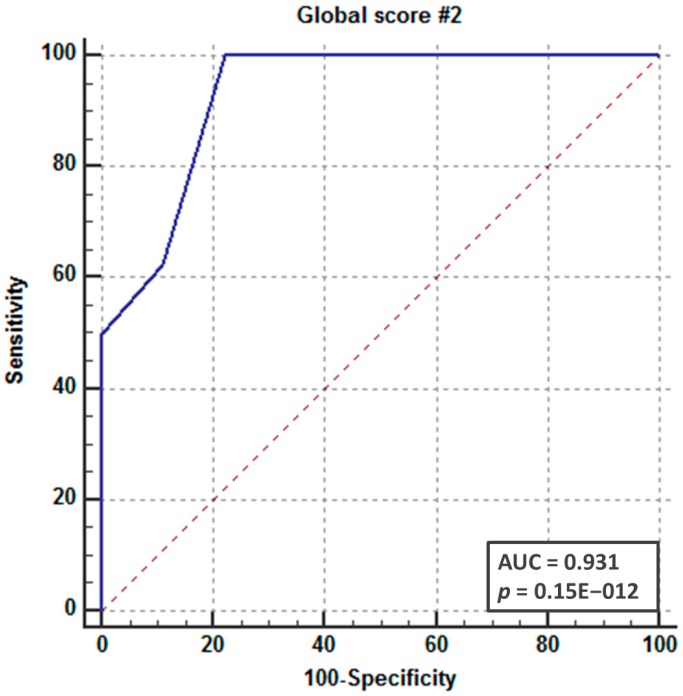
Area under the curve of Partial Score Pattern C. The optimal cut-off value was 2, corresponding to one subsite with Pattern C signal abnormalities.

Using “Partial Score Pattern C” as a dependent variable and histopathological status as the reference, an AUROC curve of 0.931 (CI 95%, 0.698–0.997) was obtained (Figure 6), using 2 as the best cut-off (100% sensitivity and 78% specificity). This means that if at least one of the considered structures in the PLC is characterized by Pattern C (Score 2), a neoplastic origin of arytenoid fixation is suggested (to be confirmed upon histopathological examination). On the other hand, if these changes are absent, neoplastic involvement is very unlikely.

## 4. Discussion

CT is widely used as a first-choice technique in the staging and treatment planning of laryngeal SCC. However, with this technique the assessment of the PLC has some intrinsic limitations. Even after contrast administration, in fact, the density of the tumor overlaps with that of adjacent soft tissues, making discrimination between normal and invaded structures difficult. This is explained by the fact that adipose tissue, which in other subsites of the larynx (like the pre-epiglottic space and upper PGS) facilitates the definition of the deep extent of the tumor, is usually under-represented in the PLC at the glottic level [7,8]. In addition, although spatial resolution of CT allows detection of subtle anomalies of the cortical bone of ossified cartilages, bone marrow alterations are difficult to spot and the diagnostic value of ancillary findings, like arytenoid sclerosis, is limited [9]. This may be improved by dual-energy CT, but the sensitivity remains low compared to MR [10,11].

In our study, we evaluated a small but extremely homogeneous retrospective cohort of patients affected by cT3 glottic SCC with arytenoid fixation as indicated by preoperative videolaryngoscopy. Due to the key role of clinical staging, preoperative video-recordings were further reviewed upon inclusion in the study to confirm the presence of true CAU fixation (cT3). Our results showed that accurate MR analysis may predict tumor infiltration of CAU and, more generally, of PLC, distinguishing it from inflammatory changes. In fact, if a state-of-the-art technique (including surface coils, adequate spatial resolution, and special sequences for uncooperative patients) is used, the morphologic and signal changes of all structures can be appropriately identified.

Interestingly, Pattern B was never associated with neoplastic infiltration. Notably, although the combination of a high-T2 signal and the absence of DWI restriction is an accepted pattern of inflammation, in our study the detection of these signal changes actually corresponded to the complete absence of neoplastic cells even on a microscopic scale, and not to a mix of abundant inflammatory infiltrate and sparse neoplastic cells. Furthermore, Pattern C was mainly associated with tumor infiltration (with the exception of two false positives in the present series), as described in the literature in recurrences after transoral laser microsurgery [12].

To the best of our knowledge, no study has focused on a similar topic with which we could compare our results. In view of its excellent negative predictive value, MR may represent a useful tool for differentiating patients with arytenoid immobility due to neoplastic involvement (cT3/pT3) from those in which arytenoid fixation has inflammatory causes (cT3/pT2). Indeed, this can greatly influence treatment choice, favoring conservative approaches aimed at preserving laryngeal function, as well as requiring elective management of neck lymph nodes or predicting postoperative adjuvant treatments [4,13,14,15,16].

However, the present study has some drawbacks that should be taken into consideration. First of all, it involved a retrospective analysis carried out on a small subset of clinically homogeneous patients who were evaluated, treated, and histopathologically analyzed by the same multidisciplinary team. Due to the retrospective nature of the study, histopathological analysis was not performed in a way to allow a truly comprehensive subsite by subsite matching. In the future, a structured reporting model will be developed in order to prospectively collect a higher number of clinical, histological, and radiological details. This could allow for further prognostic subcategories to be identified and for the development of novel, personalized treatment approaches. Nevertheless, we strongly believe that a detailed MR analysis of CAU and PLC neoplastic involvement represents an essential step in patient work-up to guide treatment selection towards surgical options like transoral laser microsurgery, OPHLs, and TL or organ preservation protocols.

## 5. Conclusions

This retrospective analysis confirms the possibility of preoperatively assessing, with good diagnostic accuracy, the actual PLC involvement in cT3 laryngeal SCC using state-of-the-art MR protocols and surface coils. A detailed evaluation of the three-dimensional local extent of disease within the larynx will definitely allow for the fine-tuning of the therapeutic approach, balancing the need for more aggressive treatments in the subset of real cT3/pT3 patients with PLC involvement, and sparing undue therapeutic morbidity to those with less critical local extension (cT3/pT2).

## Figures and Tables

**Table 1:** Baseline patient, tumor, and surgical procedure characteristics. Legend: OPHL, open partial horizontal laryngectomy; TL, total laryngectomy; ND, neck dissection.

**Mean Age** **(range)**	64.9 years (45–82) years
**Gender**	13 M, 4 F
**cT**	17 cT3
**cN**	11 cN0 6 cN+
**pT**	13 pT3 4 pT4a
**pN**	8 pN0 2 pN2a 5 pN2b 2 pN2c
**Surgery**	10 OPHL 7 TL
**ND**	10 bilatera l7 unilateral

**Table 2:** Magnetic resonance (MR) signal changes in PLC subsites. Legend: POST, posterior; VM, vocal (thyro-arytenoid) muscle; PGS, paraglottic space; ARY, arytenoid cartilage; CRIC LAM, cricoid lamina; LCAM, lateral crico-arytenoid muscle; CTM, crico-thyroid muscle; CAU, crico-arytenoid unit; PLC, posterior laryngeal compartment.

ID	POST VM	POST PGS	INF PGS	ARY	CRIC LAM	LCAM	CTM	Overall Score	Partial Score of Pattern B	Partial Score of Pattern C	Histological CAU/PLC Infiltration
**1**	1	0	0	0	0	0	0	**1**	**1**	**0**	**0**
**2**	1	1	1	0	0	1	0	**4**	**4**	**0**	**0**
**3**	2	2	0	0	0	0	0	**4**	**0**	**4**	**0**
**4**	0	1	1	0	0	1	0	**3**	**3**	**0**	**0**
**5**	1	1	0	0	0	1	0	**3**	**3**	**0**	**0**
**6**	2	0	2	0	2	2	2	**10**	**0**	**10**	**1**
**7**	0	1	1	0	0	1	0	**3**	**3**	**0**	**0**
**8**	2	1	1	0	0	2	0	**6**	**2**	**4**	**1**
**9**	2	2	2	1	0	2	0	**9**	**1**	**8**	**1**
**10**	2	2	2	1	0	2	1	**10**	**2**	**8**	**1**
**11**	2	1	1	1	0	1	1	**7**	**5**	**2**	**1**
**12**	2	1	0	0	0	0	0	**3**	**1**	**2**	**0**
**13**	1	0	0	0	0	0	0	**1**	**1**	**0**	**0**
**14**	0	0	0	0	0	0	0	**0**	**0**	**0**	**0**
**15**	2	0	0	0	0	1	0	**3**	**1**	**2**	**1**
**16**	2	2	1	2	0	1	0	**8**	**2**	**6**	**1**
**17**	2	1	0	0	0	0	0	**3**	**1**	**2**	**1**
